# Invasive lobular carcinoma with extracellular mucin as a distinct variant of lobular carcinoma: a case report

**DOI:** 10.1186/1746-1596-7-91

**Published:** 2012-08-06

**Authors:** Hacer Haltas, Reyhan Bayrak, Sibel Yenidunya, Dilek Kosehan, Meral Sen, Kayihan Akin

**Affiliations:** 1 Department of Pathology, Fatih University School of Medicine, No:57 06510, Ankara, Turkey; 2 Department of Radiology, Fatih University School of Medicine, No:57 06510, Ankara, Turkey; 3 Department of General Surgery, Fatih University School of Medicine, No:57 06510, Ankara, Turkey; 4 Fatih Üniversitesi Tıp Fakültesi, Alparslan Türkeş cad, No:57 06510, Ankara, Turkey

## Abstract

The differences between invasive lobular and ductal carcinomas affect the diagnostic and therapeutic management for patients with breast cancer. In most cases, this can be accomplished because of distinct histomorphologic features. However, occasionally, this task may become quite difficult, in particular when dealing with the variants of infiltrating lobular carcinoma. Lobular carcinoma has been considered a variant of mucin-secreting carcinoma with only intracytoplasmic mucin. The presence of extracellular mucin is a feature of ductal carcinoma. Herein is presented a case of lobular carcinoma with extracellular and intracellular mucin in a 43-year-old female patient, and confirmed by immunohistochemistry. Up to the present, infiltrating lobular carcinoma displaying extracellular mucin has not been described in the literature except two case.

**Virtual slides:**

The virtual slide(s) for this article can be found here: http://www.diagnosticpathology.diagnomx.eu/vs/1839906067716744

## Introduction

The majority of invasive breast carcinomas are categorized as ductal carcinoma. Invasive lobular carcinoma (ILC) is the second most common histological type of breast carcinoma, accounting for approximately 5%–15% of all invasive breast cancers [1,2]. Classical ILC, by definition, is a low-grade tumor with little or no nuclear atypia and a low mitotic rate. ILCs are characterized by cytologically uniform cells with round nuclei and inconspicuous nucleoli, as well as discohesive architecture with a linear or non-linear growth pattern [3-6]. Lobular neoplasia and infiltrative lobular carcinoma may produce intracellular mucin. Tumor cells may appear in signet ring shapes owing to distension with mucus. Extracellular mucin secretion is known as a feature of ductal carcinoma [4].

Herein, we present a case of lobular carcinoma with extracellular mucin and signet ring component. Up to the now, only 2 cases of mammary invasive lobular carcinoma with extracellular mucin have been described in the English written literature [7,8].

### Case report

**Figure 1:**
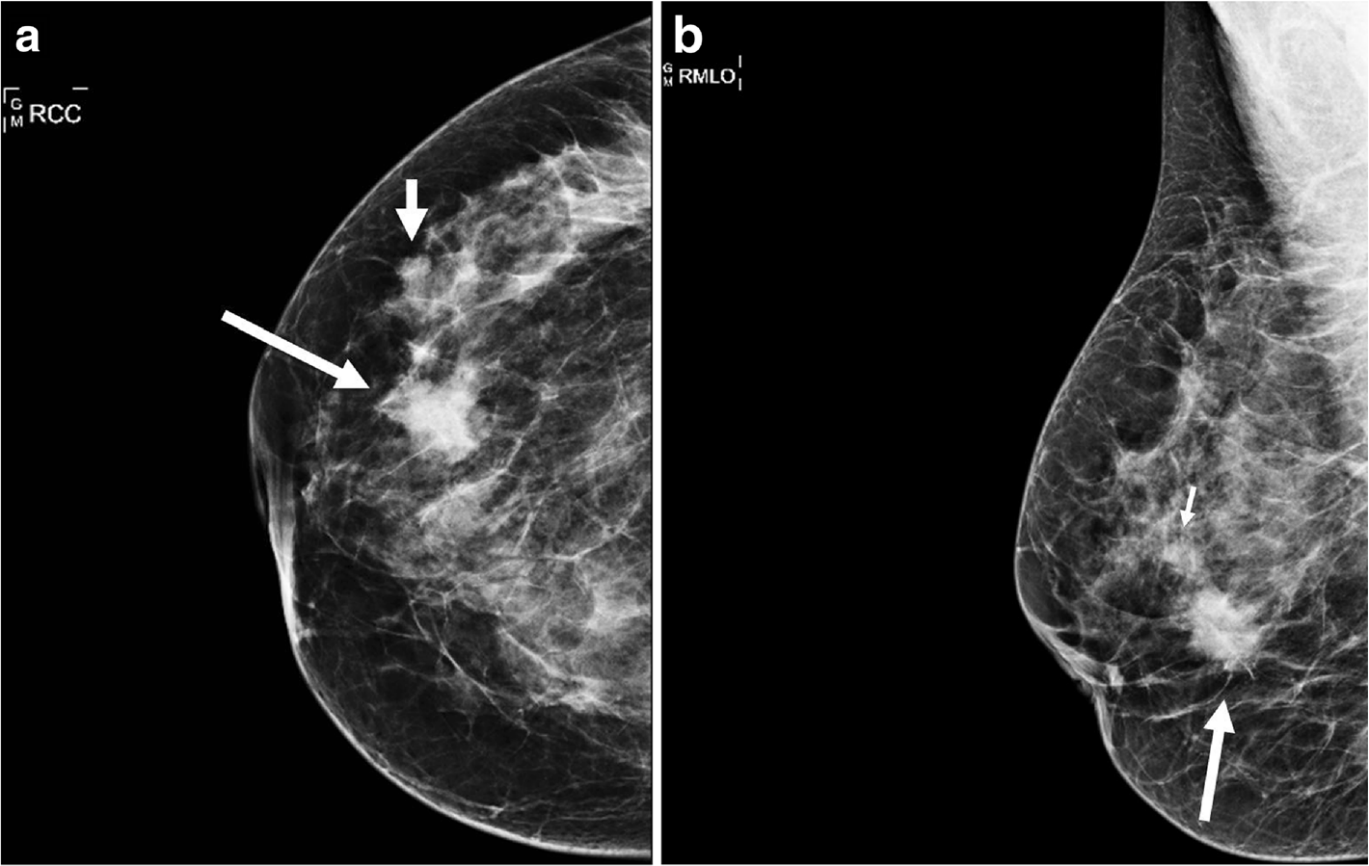
**A spiculated, irregular mass lesion (long white arrows) measuring 2.5x2 cm, located at mid-outer quadrant causing retraction of areola-nipple complex and skin thickening is demostrated on CC (a) and MLO (b) mammographies of the right breast.** A secondary lobulated satellite lesion measuring about 1 cm located superolaterally of the bigger mass was also detected (a-b, short white arrows).

A 43-year-old premenopausal woman, who had no family history of breast cancer, presented with a mass in the right breast. No axillary adenopathy was detected upon examination. A vague palpable mass was identified in the 8 o’clock region of the right breast. The palpable mass was confirmed with mammographic and ultrasonographic findings. Two lesions were detected on mamography. A primary spiculated, irregular, radiodense mass lesion measuring 2.5x2 cm, located at mid-outer quadrant of the right breast causing retraction of areola-nipple complex and skin thickening was detected on craniocaudal (CC) and mediolateral (MLO) projection mammographies of the patient. BI-RADS category was assessed to be 4 C. A secondary radiodense lobulated lesion measuring about 1 cm in diameter, located superolaterally of the bigger mass, was also detected and presumed to represent a satellite lesion. A hypoechoic, spiculated solid lesion with posterior acoustic shadowing located at 8–9 radiant at the edge of areola and a second hypoechoic solid lesion located at 9 radiant 2 cm away from areola was detected with ultrasonography consistent with mammographic findings (Figure 1a,b).

No additional abnormality was detected in the left breast. A subsequent diagnostic biopsy revealed an invasive lobular carcinoma with extracellular mucin. The patient underwent modified radical mastectomy with ipsilateral axillary clearance.

The resected tissue was fixed in 10% formalin and embedded in paraffin. Three-micrometer-thick sections were cut and stained with H&E. Histochemical stains for Mucicarmine and Alcian-Blue were used to confirm the mucin production and its localization.

Further analysis was performed using the streptavidin – biotin – immunoperoxidase technique. Immunohistochemistry for E-cadherin (clone: 36B5, Neomarkers, ready to use), Estrogen receptor (clone: SP1 Neomarkers, ready to use), progesteron receptor (clone: SP2 Neomarkers, ready to use), HER2/neu (clone: E2-4001 + 3b5, Neomarkers, ready to use) chromogranin A(clone LK2H10+PHE5, Neomarkers, ready to use), synaptophysin (clone:SYP02, Neomarkers, ready to use) were performed. The reaction product was visualized by aminoethylcarbazole (AEC) chromogen (Thermo scientific, Fremont, USA)) and counterstained with Mayer’s haematoxylin.

**Figure 2:**
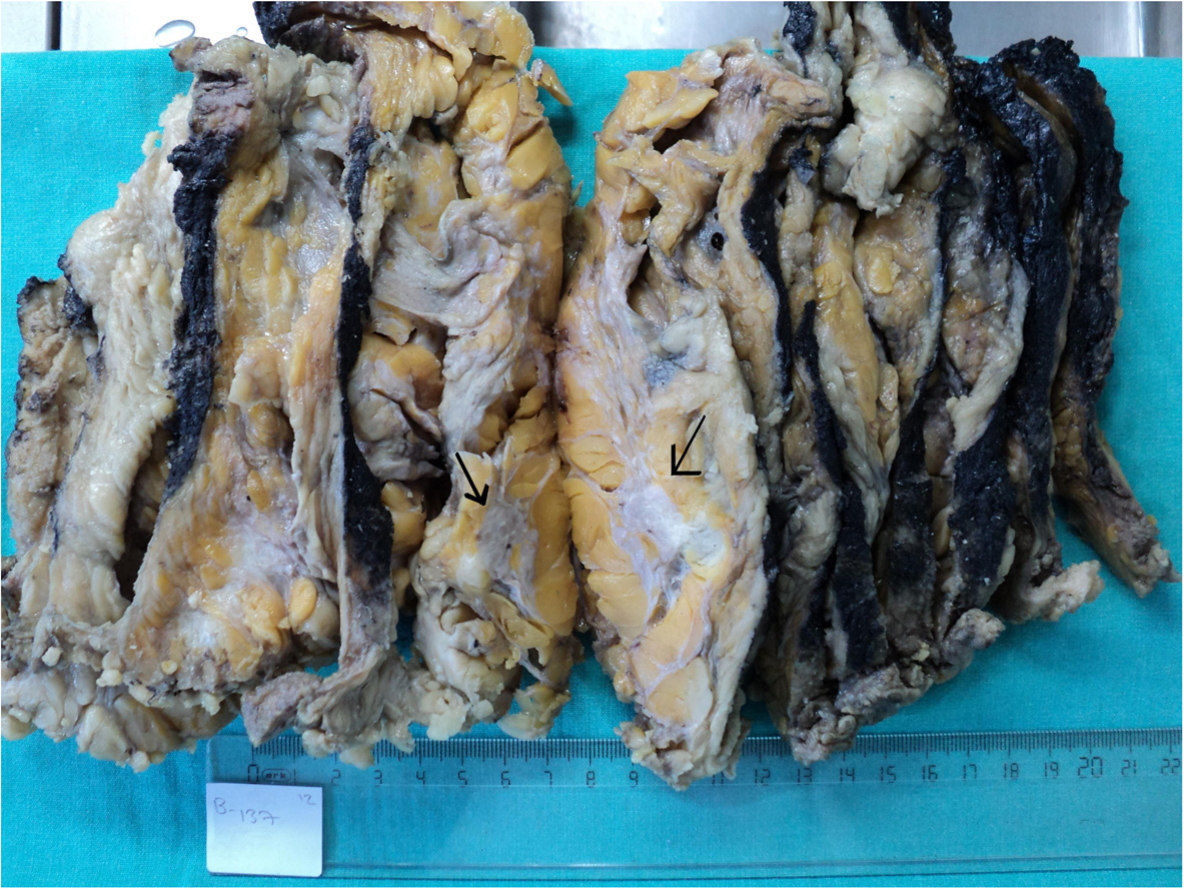
**Cut surface of the gross specimen shows irregular shaped, grey white tumor.** The tumor has the largest size of tumors.

On gross examination, two separate solid lesions, measuring 2.5 cm and 0.5 cm in maximal dimension with an intervening distance of 1 cm were identified and the tumors were located below nipple and areola complex (Figure 2). A third tumor mass measured 1x0.8x0.8 cm was observed close to the axillary region. The size of the largest invasive carcinoma was used for T classification. A cross section of the masses showed a grey white solid mucinous area. There was retraction of nipple and the skin over the lump was normal. Nineteen axillary lymph nodes were isolated.

**Figure 3:**
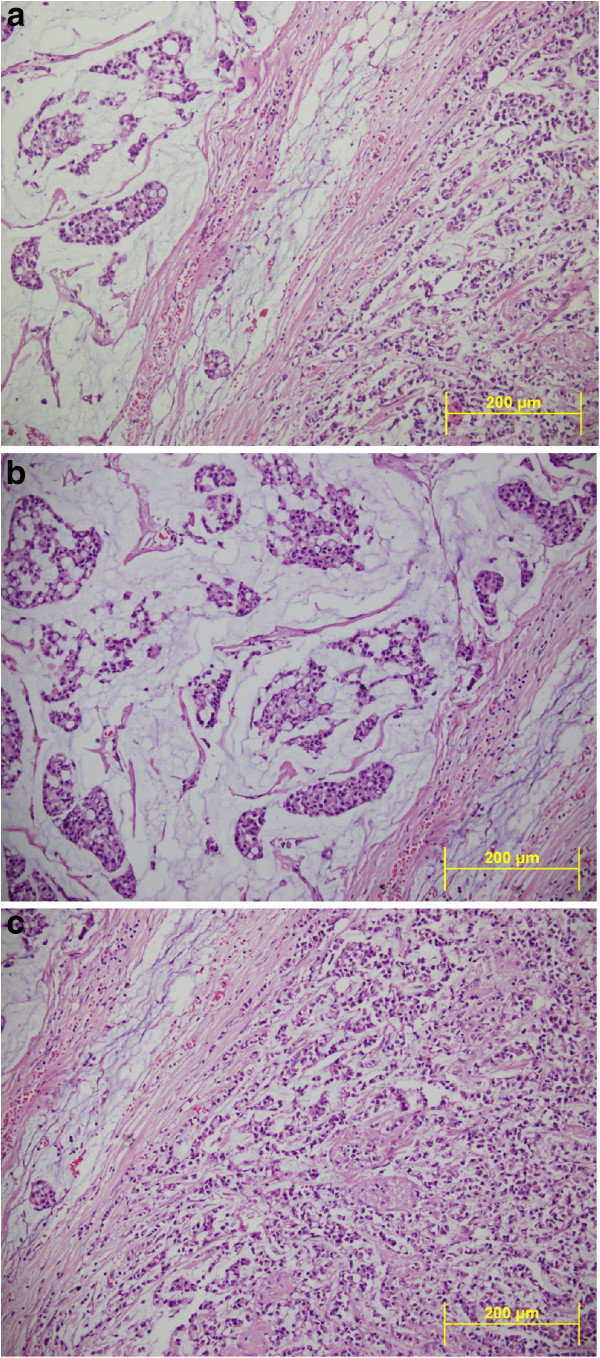
**a) Lobular carcinoma of breast with extracellular mucin showing typical single cell infiltration and groups of tumor cells floating in extracellular mucin.** (HEX200) **b**) Note that abundant extracellular mucin accumulates around invasive tumor cells (HEX200) **c**) Microscopic picture showing typical single cell infiltration of the stroma and dyshesive pattern of lobular carcinoma. (HEX200).

**Figure 4:**
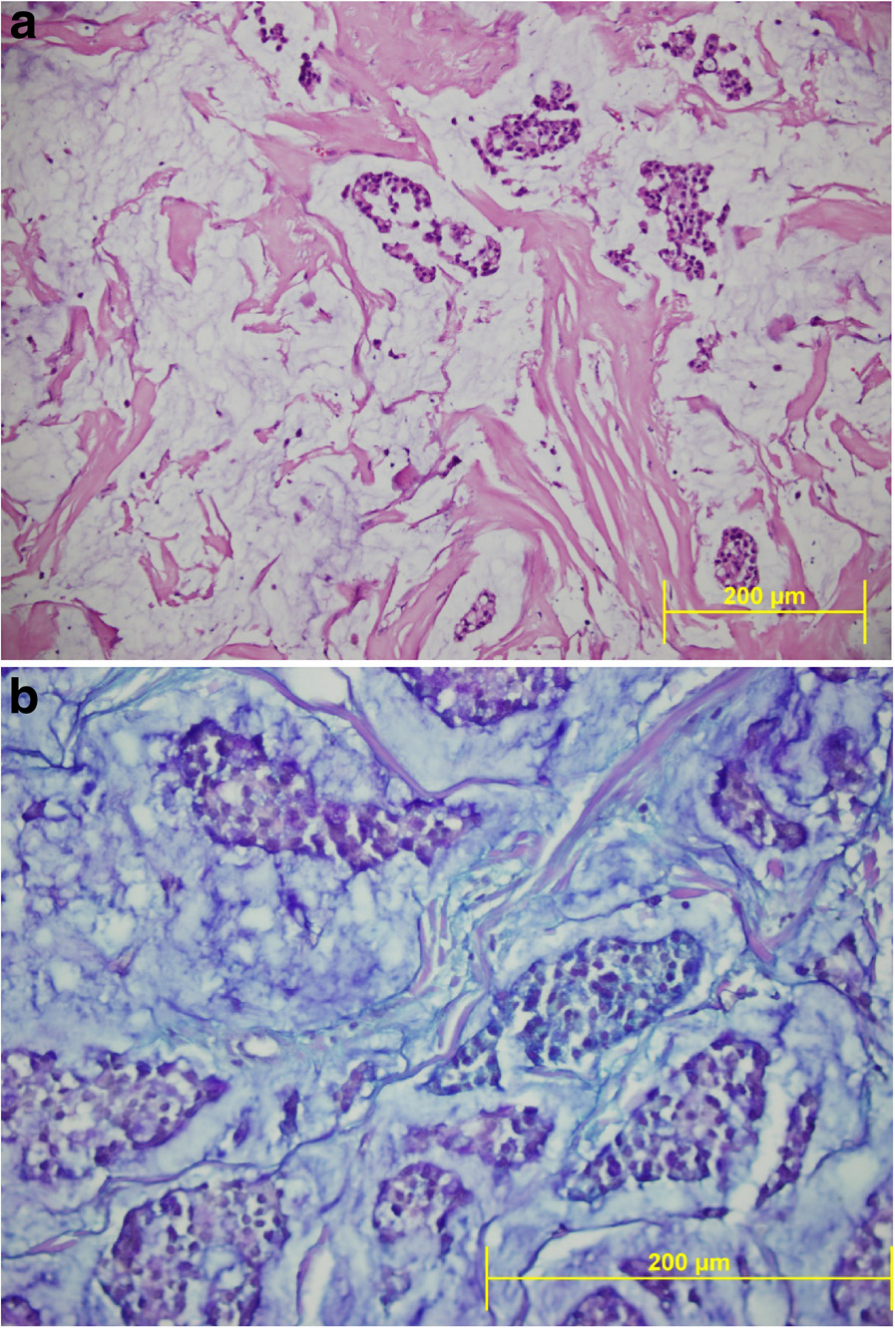
**a)Hematoxylin and eosin staining showed a lobular carcinoma with malignant mucin-filled epithelial cells floating free in mucinous pools.** (HEX200). **b**) Alcian blue/periodic acid Schiff stains showed epithelial groups floating in mucin, which stains blue. (PAS-ABX400).

**Figure 5:**
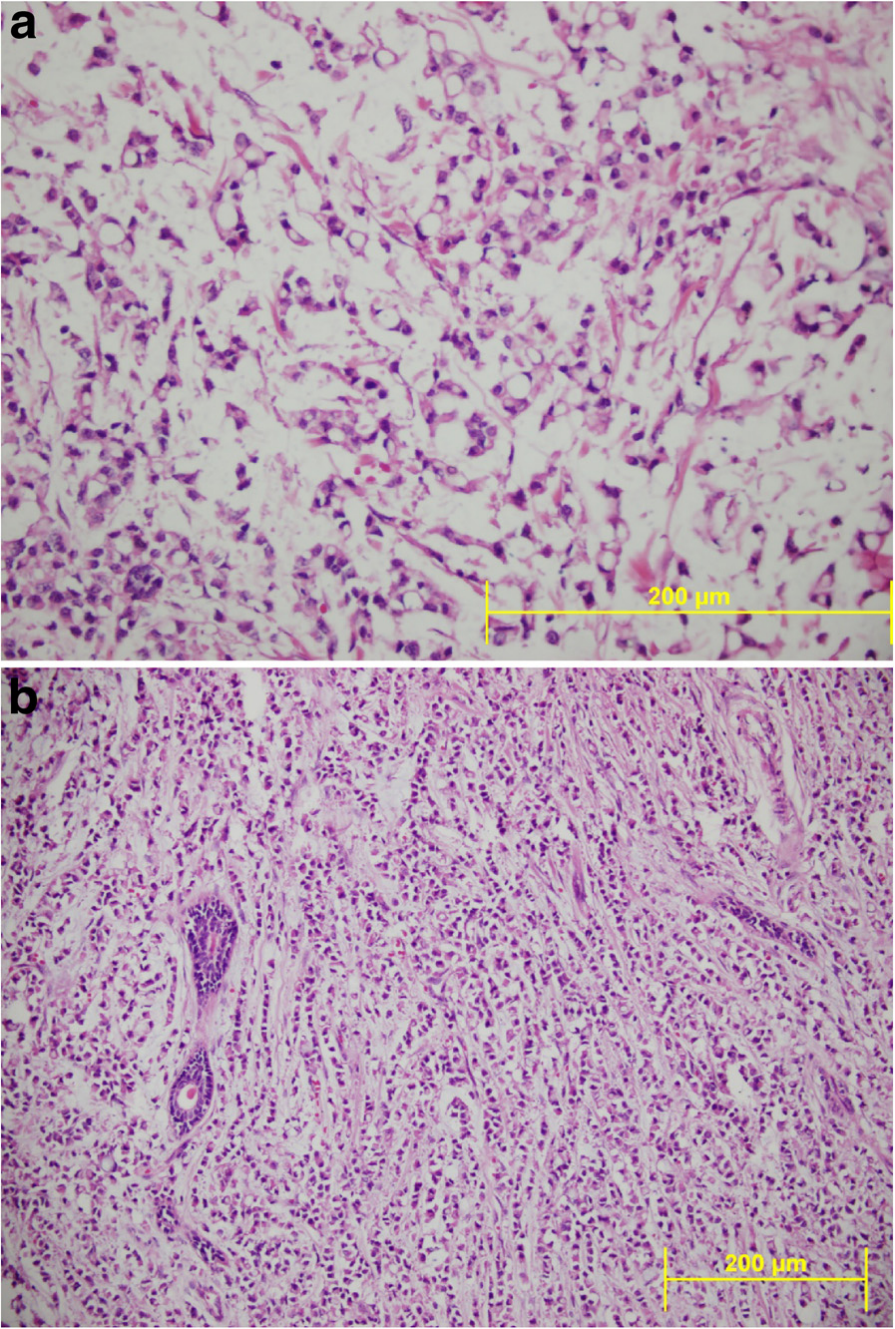
**a)Signet ring cells with distended bubbly cytoplasm and distinct intracytoplasmic lumens and abundant extracellular mucin were seen.** (HEX400) **b**) Tumor showing indian-file pattern in some area. (HEX200).

**Figure 6:**
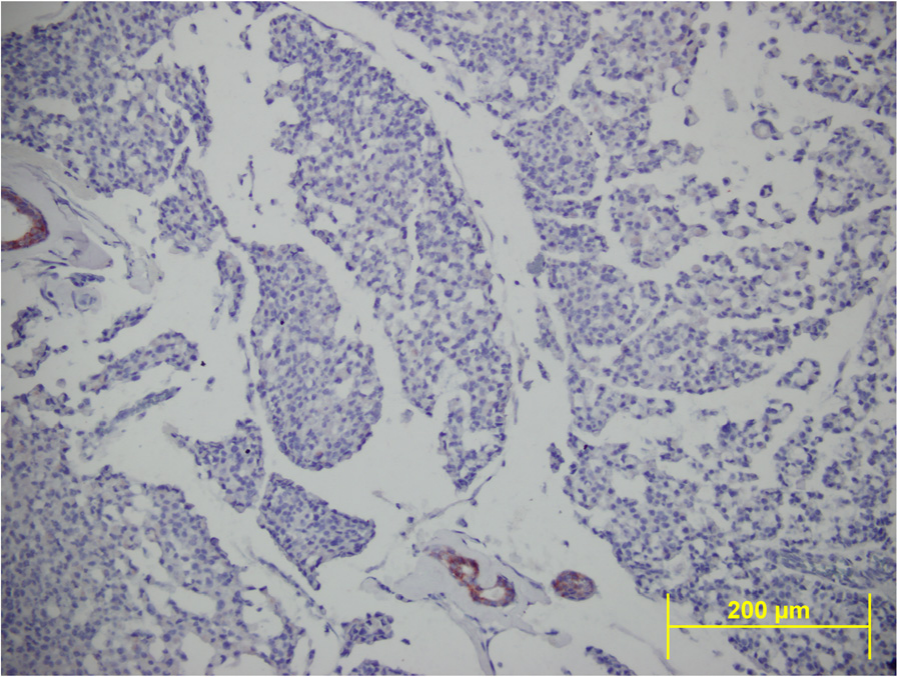
**Immunohistochemical stain showed absence of membranous E-cadherin staining.** The duct which is a positive internal control seen in the image (E-cadherin x200).

Microscope examination showed that abundant extracellular mucin was accumulated around solid tumor cells. In the peripheral areas, morphology of classic lobular carcinoma was observed (Figure 3). Mucicarmine and, PAS-Alcian-blue demonstrated the presence of intracellular and extracellular mucin (Figure 4). The tumor cells were small to medium in size, relatively uniform and round, with small nucleoli and scant to moderate amount of cytoplasm. Signet ring cells with intracellular lumina were also present (Figure 5). These coexisted with lobular carcinoma in situ. No lympho vascular invasion was observed. No nipple or skin involvement was present. Only one axillary lymph nodes were involved with tumor cells which were histologically identical with those in the breast tumor. The tumor was staged as T2N1M0 and was estrogen receptor (ER) and progesterone receptor (PR) positive. Immunohistochemically, HER2/neu and E-cadherin were found negative in the carcinomatous cells (Figure 6). Chromogranin A and Synaptophysin, used to exclude neuroendocrine differentiation, were negative.

Informed consent was obtained from the patient.

## Discussion

Invasive lobular carcinoma is a distinct type of breast carcinoma based on its characteristic histological pattern. It is more frequently hormone-receptor positive, displays a higher incidence of synchronous, contralateral primary tumors, more frequently presents with multicentric disease, and metastasizes to distinct sites such as the meninges, serosa, and retroperitoneum [4-6]. These tumors arise from the lobular and terminal duct epithelium. They can occur throughout the entire age range of breast carcinoma in adult women and usually constitutes 5-15% of carcinomas. Besides the classical invasive lobular type, other variant forms are also seen [1,3,4]. Histologically, the classical type of ILC is characterized by dyshesive cells with small nuclei, linear arrangements of cells infiltrating the stroma between collagen fascicles forming so-called ‘Indian files’ and low mitotic activity. Lobular carcinoma, both in situ and infiltrating, is a tumor that secretes acidic mucosubstances, that are intracellular in location [3]. When the secretion is prominent, the cells have a signet ring configuration [4]. The well-described variant ILCs include solid, alveolar, pleomorphic, tubulolobular, signet ring, and mixed types [3-6].

Although generally accepted histological criteria serve to distinguish lobular from ductal carcinoma of the breast, this differential diagnosis may present a challenge in some variants of the tumors showing equivocal histological features [4]. In breast tumors, extracellular mucin production is encountered as a feature of ductal phenotype [4,6]. In our case report, lobular carcinoma with abundant extracellular mucin was detected.

It is important for pathologists to recognize invasive lobular carcinoma with extracellular mucin because of the differential diagnosis. The histological differential diagnosis of the tumor may include pure mucinous carcinoma, mixed mucinous-ductal carcinoma, mucinous carcinoma with neuroendocrine differentiation, mucinous papillary neoplasms, mucocel like tumor, and mixt carcinoma (lobular and ductal carcinoma). These tumors have ductal phenotype. The distinction is important for their prognosis and management. In the breast, E-cadherin is useful to distinguish between ductal and lobular neoplasia. Tumor cell of lobular carcinoma tends to have a loss of expression of E-cadherin. E-cadherin, a cell-cohesion protein encoded by a gene on chromosome 16q22.1, is the current marker of choice to help discriminate between lobular and ductal carcinoma [4,6]. The majority of usual ductal carcinomas express membranous E-cadherin, whereas most in situ and invasive lobular carcinomas, both classic and pleomorphic types, lack its expression. In our case, The tumor was composed of small clusters of neoplastic cells disposed in large pools of mucin and classical lobular carcinoma areas. The complete loss of membranous E-cadherin in all areas of the tumor was detected. Ductal carcinoma in situ was not detected in any part of the tumor, but lobular carcinoma in situ was observed in many areas of the tumor. Also we used Chromogranin A and Synaptophysin to exclude the neuroendocrine differentiation of the tumor, where we observed that, these markers were negative.

The majority of invasive lobular carcinomas (ILCs) express estrogen receptor (ER) and progesterone receptor (PR). HER-2 overexpression and amplification are limited essentially to invasive ductal carcinomas of intermediate to high grade. Classical lobular carcinoma does not show HER-2 overexpression or amplification [6]. Rosa and colleagues observed that the tumor did not overexpress HER2 protein in the first case of lobular carcinoma with extracellular mucin, similarly to our results [7]. On the other hand, Yu and colleagues found overexpression of HER2 protein in lobular carcinoma with extracellular mucin in their case report. They thought that this tumor was between lobular and ductal carcinomas to the overlapping morphological features as well as molecular manifestation [8]. Because number of cases of these tumors is limited, it is difficult to comment on the biological behavior and molecular profiles.

Lobular carcinoma with extracellular mucin secretion is a newly described extremely rare variant with only two cases reported in the English medical literature. Rosa and colleagues reported the first case, and Yu and colleagues reported the second case [7,8]. These two cases are summarized in Table 1. The current report is the third documented case.

**Table 1:** Summary of the reported case of lobular carcinoma with intra and extracellular mucin secretion

**Authors**	**Age**	**Site**	**Surgery**	**ER/PR**	**HER2**	**Lobular Carcinoma In situ**	**Multifocality**	**Signet ring component**	**Positive lymph nodes**	**Additional pathology**
Rosa et al., [7]	60	right breast	simple mastectomy	+/+	-	+	-	+	axillary dissection not performed	synchronous ductal carcinoma in left breast
Yu et al. [8]	65	left breast	lumpectomy	+/-	3+	+	-	+	sentinel lymp node metastasis	-
***This case,2012***	***43***	***right breast***	***modified radical mastectomy***	+/+	-	+	+	+	**axillary lymp node metastasis (1/19)**	-

In conclusion, we have reported a very rare case of lobular carcinoma with intra and extracellular mucin secretion. Extracellular mucin secretion may not be an exlusive feature of ductal phenotype.

## Competing interests

The authors declare that they have no competing interests.

## Authors’ contributions

HH, RB, SY, and MS collected the data and drafted the manuscript. HH, RB, SY carried out the gross examination and final diagnosis. DK and KA provided the radiologic imaging data. All authors read and approved the final manuscript.
